# The SHAZ! Project: Results from a Pilot Randomized Trial of a Structural Intervention to Prevent HIV among Adolescent Women in Zimbabwe

**DOI:** 10.1371/journal.pone.0113621

**Published:** 2014-11-21

**Authors:** Megan S. Dunbar, Mi-Suk Kang Dufour, Barrot Lambdin, Imelda Mudekunye-Mahaka, Definate Nhamo, Nancy S. Padian

**Affiliations:** 1 Pangaea Global AIDS Foundation, Oakland, CA, United States of America; 2 University of California San Francisco, Center for AIDS Prevention Studies, San Francisco, United States of America; 3 University of California, School of Public Health, Berkeley, United States of America; 4 Zimbabwe AIDS Prevention Program, Harare, Zimbabwe; University of Ottawa, Canada

## Abstract

Adolescent females in Zimbabwe are at high risk for HIV acquisition. Shaping the Health of Adolescents in Zimbabwe (SHAZ!) was a randomized controlled trial of a combined intervention package including life-skills and health education, vocational training, micro-grants and social supports compared to life-skills and health education alone. SHAZ! was originally envisioned as a larger effectiveness trial, however, the intervention was scaled back due to contextual and economic conditions in the country at the time. SHAZ! enrolled 315 participants randomly assigned to study arm within blocks of 50 participants (158 intervention and 157 control). The intervention arm participants showed statistically significant differences from the control arm participants for several outcomes during the two years of follow up including; reduced food insecurity [IOR = 0.83 vs. COR = 0.68, p-0.02], and having their own income [IOR = 2.05 vs. COR = 1.67, p = 0.02]. Additionally, within the Intervention arm there was a lower risk of transactional sex [IOR = 0.64, 95% CI (0.50, 0.83)], and a higher likelihood of using a condom with their current partner [IOR = 1.79, 95% CI (1.23, 2.62)] over time compared to baseline. There was also evidence of fewer unintended pregnancies among intervention participants [HR = 0.61, 95% CI (0.37, 1.01)], although this relationship achieved only marginal statistical significance. Several important challenges in this study included the coordination with vocational training programs, the political and economic instability of the area at the time of the study, and the difficulty in creating a true standard of care control arm. Overall the results of the SHAZ! study suggest important potential for HIV prevention intervention packages that include vocational training and micro-grants, and lessons for further economic livelihoods interventions with adolescent females. Further work is needed to refine the intervention model, and test the impact of the intervention at scale on biological outcomes.

**Trial Registration:**

ClinicalTrials.gov NCT02034214

## Introduction

In Zimbabwe, as in much of Sub-Saharan Africa, prevalence of Human Immunodeficiency Virus (HIV) is high at 15%[1], and effective HIV prevention, care and treatment remains one of its most pressing problems. Adolescents (aged 15–19) in Zimbabwe experience a particularly high burden of HIV, with females twice as likely as males to be HIV infected (6% vs 3%, respectively) [1]. Over the last decade, Zimbabwe has faced a severe economic and political crisis, fuelled by a government-led land redistribution program that, by 2008, had resulted in a collapse of the agricultural sector, skyrocketing inflation, massive unemployment, and widespread civil unrest [2],[3]. This overarching context has exacerbated a situation in which adolescent women already face highly constrained access to educational and economic opportunities, and health services [4]–[9], and has likely helped to fuel the rising epidemic among girls and adolescent women.

Research has shown that the inequitable burden of HIV among adolescent women is driven, at least in part, by structural factors, defined as environmental, social and economic barriers to an individual's HIV prevention and health-seeking or treatment behavior [10],[11]. Specific factors that have been linked to HIV risk among adolescent women in sub-Saharan Africa include poverty and income inequality; lack of educational opportunities; gender inequalities in social structures and relationships; and gender-based or intimate partner violence [4]–[6,12–15]. Within this context, in addition to early marriage, selling sex becomes one of the few means available to supplement or generate income. While few may result to full-time sex work, many adolescent women trade sex for food, mobile air time and other expenses [16], a phenomenon observed throughout Zimbabwe and the sub-Saharan African region [17]–[28]. The associated sexual risks, including exposure to violence, have been linked to HIV infection [29].

In an effort to address these so-called structural drivers of HIV, a new generation of research and programming has emerged to promote HIV prevention among young and adolescent women [12]. Such efforts have included interventions to increase gender equity, provide conditional and non-conditional cash transfers, and offer microcredit or other livelihoods opportunities [12,14,30]–[39]. For instance, IMAGE (Intervention for Microfinance and Gender Equity) merged a curriculum of gender equity and HIV/AIDS education with microfinance for women in South Africa. While no reduction in HIV incidence was observed from its community randomized controlled trial (RCT), results demonstrated effects on related outcomes such as increased assets [38], a 55% reduction in intimate-partner violence [38], and increased condom use [39]. In Zimbabwe, an RCT of 25 primary schools studied the effects of providing school fees to orphaned girls. HIV was not directly measured, but the study found an 82% reduction in school drop-out and marriage rates after two years, and more equitable gender attitudes [37]. Recently, an RCT of a cash transfer (CT) program among 1,289 girls attending school in Malawi found lower odds of HIV (OR 0.36, 95% CI 0.14–0.91) and HSV-2 (OR 0.24, 95% CI, 0.09–0.65) among those who received CTs compared to those that did not [35].

While these studies provide reason for optimism about economic structural interventions for women and girls, most efforts have neglected those arguably at greatest risk: adolescent women participants who have completed or left school. As Gibbs et al point out in a recent review of structural interventions [12], microcredit and gender transformative interventions tend to work best among mature women, and while cash transfers have shown promising effects for school-based adolescents, these approaches leave out those who have completed school or who do not have the interest or ability to continue their education – even when school fees or other support is provided.

However, finding effective approaches for this group is challenging. In an evaluation of the Tap and Reposition of Youth (TRY) program, which rolled-out microcredit with business training and mentoring among out-of-school adolescent women (aged 16–22 years) in Kenya, marginal improvements were seen in gender attitudes, but not in SRH knowledge, and there was a 66% drop out rate. In the formative phase of the SHAZ! (Shaping the Health of Adolescents in Zimbabwe) [40], the authors of this paper tested a combined microcredit and life skills intervention among 50 out-of-school female orphans aged 16–19 years in Zimbabwe. While HIV knowledge increased (16% vs 38%, p<0.001), no statistically significant changes were reflected in future aspirations, sexual activity, or condom use, and at 6 months, only 20% had begun repayment. Furthermore, the economic situation in Zimbabwe (as described above) ultimately proved microcredit to be unfeasible, and potentially harmful, as it led some to engage in new livelihoods strategies, including cross-border trading, that placed them at increased risk of sexual harassment and violence [40]. Subsequent improvements in SHAZ!, the topic of this paper, have addressed these findings through the use of a combination approach that applies economic alternatives that focus on training and support, and economic opportunities that do not demand repayment.

### Shaping the Health of Adolescents in Zimbabwe: SHAZ! Pilot Intervention

SHAZ! began with formative work in 2000,which showed that poverty and gender inequalities increased adolescent women's HIV risk [41], and that that maternal orphans were most vulnerable [42]. Results of a formative phase evaluation of a micro-credit intervention ((4), also described above) led to a radical re-design of the model, based not only on the pilot data, but also on the concepts of agency and gendered poverty as described in Naila Kabeer's Theory of Women's Empowerment [43],[44]. This theory posits that empowerment is a process by which one develops increased access to resources (informational, social and economic) and greater agency (or control), ultimately improving capabilities, or the capacity to effect outcomes in one's own life. The resulting SHAZ! Pilot Intervention – the evaluation of which is described in this paper – represents a radical modification of SHAZ!, from micro-credit to livelihoods training and micro-grants. The redesign was guided by input from young women and community advisors to find an appropriate and effective structural intervention approach addressing the needs of out-of-school adolescent girls and women, in this challenging political, social and economic context.

**Figure 1:**
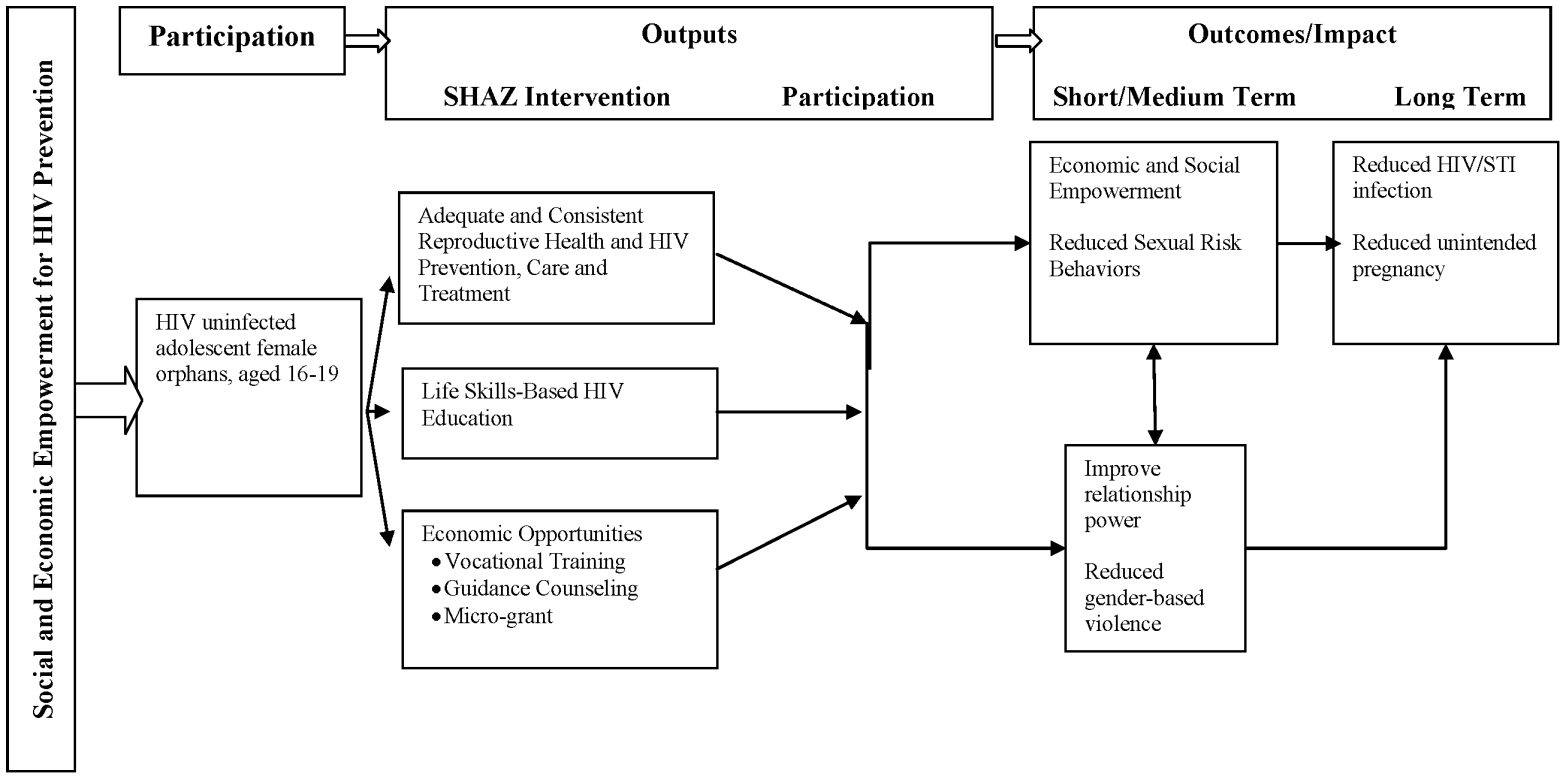
SHAZ! Theoretical Framework.

Figure 1 depicts how we hypothesized the SHAZ! Pilot Intervention would improve HIV and sexual and reproductive health (SRH) outcomes among our target population. The program included four main activities (described in detail below) to comprise the full intervention: (1) providing adequate and consistent HIV and sexual and reproductive health services; (2) conducting life skills-based HIV education; (3) improving economic opportunities through vocational training, guidance counseling and a micro-grant; and (4) integrated social support. We hypothesized that these activities would work together to increases knowledge, and improve social and economic indicators; enabling participants to reduce risky behaviors and optimize healthy ones. We further hypothesized that taken together, these improvements will lead to reduced HIV acquisition, and unintended pregnancy.

In detail, intervention components included:

#### Reproductive Health Services (All participants)

All participants were provided a health screening at every study visit and were treated for treatable STIs and minor ailments. They received condoms, and contraceptive pills or injectables free upon request. Participants who tested positive for HIV were referred to local clinics, where the study team assisted with ART registration including payment for CD4 tests required for enrolment.

#### Life skills education and home-based care training (All participants)

The life skills curriculum drew upon Stepping Stones [36]and CDC-Zimbabwe Talk Time [45], developed with input from the target population. It consisted of 14 modules delivered to groups of 25over 4–6 weeks on: HIV/STI and reproductive health; relationship negotiation; strategies to avoid violence; and identification of safe and risky places in the community. Participants also attended a six-weeks-long home-based care training conducted through Red Cross Zimbabwe to gain skills around safely caring for people living with HIV.

#### Livelihoods (Intervention participants only)

The Livelihoods intervention consisted of financial literacy education and a choice of vocational training at local training institutes. Courses were 6-months-long, conducted in English, with a practical and a theoretical component. In spite of encouragement to venture outside of accepted gender norms, the most popular courses were hairdressing, garment-making, and receptionist/secretarial and nurse-aid training. Participants who successfully completed a vocational training developed business plans that were supported with a micro-grant in the form of capital equipment, supplies or additional training. The resources purchased with these micro-grants were valued at $100 US dollars or less per participant.

#### Integrated Social Support (ISS) (Intervention participants only)

Integrated social support undergirded the livelihoods component. ISS consisted of guidance counselling provided by trained staff to help participants navigate challenges, and self-selected adult mentors.

The SHAZ! Pilot Intervention evaluation was originally envisioned as a large-scale trial of 1,000 participants, powered to detect changes in HSV-2 incidence as a proxy for HIV. However, during the scheduled time for intervention roll-out in 2005, Zimbabwe experienced major economic and political challenges that forced delays and a scaling-back of the study. As described briefly above, these changes included a national land-reform initiative that included political violence and which destroyed Zimbabwe's economic productivity. The land reform measures also lead to massive inflation which, at its peak in 2008, was estimated at 6.5 sextillion percent [46]. In 2005, Zimbabwe also instituted operation *murambatsvina (or drive out filth)*a large-scale Zimbabwean government campaign to forcibly clear slum areas across the country. The campaign began in May of 2005, and lasted for several months, including targeting of the community where our intervention study was to take place. According to United Nations estimates, the operation affected at least 700,000 people directly through loss of their home or livelihood, and/or internal displacement, and indirectly affecting approximately 2.4 million people [47]. Given these developments, we felt it was critical to assess first, through a smaller scale Pilot Intervention study, whether the intervention would be feasible, and potentially effective in this volatile context. We therefore powered the study to detect effects on proximal (rather than HIV) outcomes. The main aims of the Pilot Intervention Study were to: assess: a) the feasibility of recruiting and retaining a cohort of adolescent female orphans who adhere to the intervention; b) changes in behavioural and structural risk factors; and c) trends in HIV and HSV-2 incidence, and unintended pregnancy.

## Methods

### Ethics statement

This research was approved by the Medical Research Council of Zimbabwe, the committee for the protection of human subjects at the University of California San Francisco and the institutional review boards at RTI International and Pangaea Global AIDS. All participants gave written informed consent to participate in the study.

### Study Design

SHAZ! II was an individual RCT assessing feasibility and potential efficacy. We compared the full intervention (e.g. all the components described above) to a control condition of life-skills (including home-based care training)and reproductive health services alone. Neither participants nor study staff were blinded to study arm assignment. After the final follow up visit, control participants who successfully completed 80% of training sessions and study visits were supported to enrol in an equivalent vocational training course. A qualitative component accompanied the quantitative RCT, the findings from which are outside the scope of this paper and will be presented elsewhere. This study was registered at Clinicaltrials.gov (reference number NCT02034214) following completion as the trial was not initially considered to meet the criteria of a clinical trial. The Consort Checklist and full protocol are available as supporting information; see Checklist S1 and Protocol S1. The authors confirm that all ongoing and related trials for this intervention are registered. Data can be made available on request to researchers who have completed human subjects training at the discretion of the University of California Committee on Human Research.

### Study Sample

A convenience sample of 315 adolescent female orphans (having lost at least one parent) aged 16 to 19 was recruited through community outreach and referrals. All eligible participants were out of school, not currently pregnant, HIV-uninfected, and living in Chitungwiza, a high density, urban area outside of Harare, Zimbabwe. Enrolment took place between February and August 2006 with the last study visit taking place in December of 2008 as scheduled. All participants gave written informed consent. The legal age of consent in Zimbabwe is 16, therefore parental consent was not required, a consenting process approved by all three of the IRBs who reviewed the protocol.

### Randomization

Eligible participants were randomized using consecutively numbered opaque envelopes containing a study arm designation. Randomization numbers were generated by the study statistician in SAS, allocated to the study in blocks of 50 to achieve balance in the life skills courses (at 25 members for each arm). Informed consent, enrolment and study arm assignment was conducted by a staff member not otherwise involved in the recruitment process.

### Data Collection

Participants were interviewed using a combination of Audio Computer Assisted Self Interviews (ACASI) to encourage the accurate reporting of sensitive sexual behaviors, and face to face interviews to ensure adequate care and referrals for participants who reported violence and/or health concerns. Data collection took place at the research clinic at baseline and every six months after for 24 months. Studystaff were trained to respond to the needs of adolescents; younger female staff were preferentially hired to improve the comfort level of participants.

### Measures

#### Structural and Sexual Risk Factors

A variety of measures was collected on structural and sexual risk factors. All measures were dichotomous, unless otherwise indicated.

1. *Economic factors* included *educational status*, indicating that the participant had completed (or not) four years of secondary school; *food insecurity*, defined as not having eaten for at least one entire day in the previous week because no food was available; and *income*, indicating that the participant had received independent income during the previous month.
2. *Social factors* included *orphan status, level of social support received, relationship power* and *experience of violence*. *Orphan status* was defined as maternal orphan (having lost a mother), paternal orphan (having lost a father) or double orphan (having lost both parents). Maternal orphan-hood was considered a sign of greater social vulnerability based on previous research [42],[48]. To measure levels of *social support received*, we adapted a measure developed in Zimbabwe that is used in orphan programming. Social support responses were assigned a value and then averaged to get a composite score that was divided into tertiles of “low” “medium” and “high” social support. The Cronbach's alpha for this measure in our population was 0.67.

*Relationship power* was measured through a modified version of the South African sexual relationship power scale [49], a measure that has been validated for use in several African contexts. Participants were allowed to report on up to three partners (e.g. a primary with two secondary partners), and questions from the scale were asked of each reported partner. Responses were coded, averaged, and divided into “High” or “Low” relationship power. To assess *experience of violence*, participants were asked if they had experienced physical violence (hitting, kicking and/or shoving), sexual violence (unwanted touching without penetration) and/or forced penetration (rape) since their last study visit. If they answered “yes” to any of these, they were coded as having experienced violence and referred to psycho-social support programs, and child protective services.

3. S*exual risk factors* included whether participants had *ever engaged in vaginal or anal sex* and if they were *currently sexually active*. Those who reported current sexual activity in the previous month were asked if they used *male or female condoms* and/or other forms of *modern contraception* (such as pills, injectibles, IUDs, implants, diaphragm and spermicides). If any modern method use was indicated, the measure was coded as “yes” regardless of specific type. Participants who reported sexual activity also were asked if they had received money, material goods, school fees or grades, food or shelter from sexual partners, and/or if they had been taken places by sexual partners, for example to the movies or out to eat. Participants were considered to have engaged in *transactional sex* if they reported that they would not have had sex with a given partner if they had *not* received these items or been taken places.
4. *Biological measures.* All participants were tested for incident *HIV* infection, using two consecutive rapid HIV enzyme-linked immunosorbent assay (ELISA) assays, in accordance with the existing standard in Zimbabwe. If both tests showed positive results, the participant was diagnosed with HIV. If both tests showed negative results, the participant was considered to be HIV-uninfected. If the two tests were discrepant, a DNA Polymerase chain reaction (PCR) determined final diagnosis. Participants were also tested for *HSV-2* infection, using standard Elisa assays; and *pregnancy*, via urine human chorionic gonadotropin (hCG) dipstick test. Pregnancy intentions were assessed using a measure derived from the London Measure of Unplanned Pregnancy [50], which has shown good reliability and validity (Cronbach's alpha  =  0.92; test-retest reliability  =  0.97). *Unintended pregnancy* was defined as any previous or biologically confirmed current pregnancy for which a participant reported not having intended or wanted to get pregnant in the month preceding.

### Statistical Methods

To check randomization, baseline factors were compared between study arms using chi-squared tests (for categorical variables) or t-tests (continuous variables). Feasibility was assessed by comparing adherence to the intervention (in terms of completed study activities) and 24 month retention.

We conducted an intent-to-treat analysis of intervention effects on economic, social and sexual risk factors – using generalized estimating equations with an exchangeable correlation structure for repeated measures – and logistic regression models to assess differences between study arms, changes over time within each study arm, and allowing interaction to assess whether changes over time *within* each arm varied *between* arms. Cox proportional hazards models compared the time to a new HIV or HSV-2 infection, or unintended pregnancy, as measured by the first positive laboratory test. To account for potential confounding of the incidence results by differential loss to follow up, biological outcomes data were also analyzed using inverse probability of censoring weighting [51]–[53].

## Results

### Participant Flow

**Figure 2:**
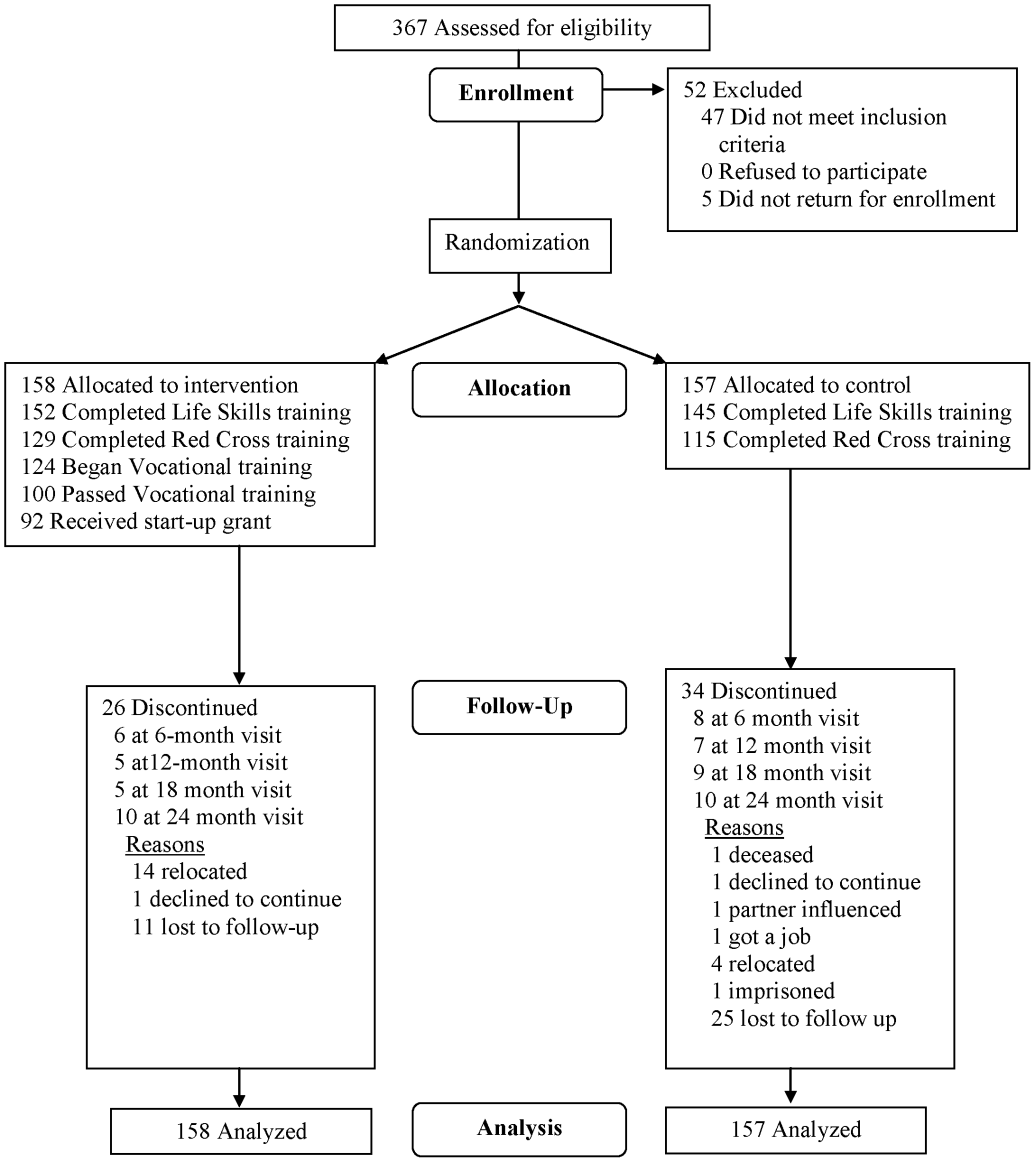
SHAZ! Study flow chart.

As presented in Figure 2, *s*creening of 367individuals resulted in 315 eligible participants. The main reason for ineligibility was a positive lab test for HIV, HSV-2 or pregnancy. Of the 315, 158 were randomized to the intervention, 157 to the control arm. Ultimately, 60 participants were discontinued from the study (26 from the intervention and 34 from the control arm)due to death, relocation, returning to formal education and partner influence. Thirty-six participants (11 in the intervention and 25 in the control arm) were lost-to-follow-up for unknown reasons.

### Baseline Characteristics and Completion of Study Activities (Table 1)

The average age of participants at study start was 18 years. The majority were unmarried (∼95%), maternal orphans (53%), and most had completed secondary school (75%). No baseline characteristics differed significantly by study arm, with the exception that more intervention participants (80%) had completed secondary school compared to control participants (70%), p = 0.03.

**Table 1:** Baseline Characteristics, Intervention Fidelity and Retention, by Study Arm.

	Total N = 315	Intervention N = 158 (%)	Control N = 157 (%)	P-value
**Demographics**				
Age, Mean (Std Dev)	18 (1.0)	18 (0.9)	18 (1.0)	0.619
Orphan status				0.204
Paternal	168 (53)	92 (58)	76 (48)	
Maternal	38 (12)	18 (11)	20 (13)	
Double	109 (35)	48 (30)	61 (39)	
Married/living with partner	11 (4)	6 (4)	5 (3)	0.742
Completed secondary school	237 (75)	127 (80)	110 (70)	0.034
**Intervention Components & Retention**				
Completed Life Skills Training, N (%)	297 (94)	152 (96)	145 (92)	0.141
Completed Red Cross Training, N (%)	244 (77)	129 (82)	115 (73)	0.075
Initiated Vocational training, N (%)		124 (79)	-	
Completed Vocational Training, N (%)		100 (63)	-	
Received micro-grant, N (%)		92 (58)	-	
24 Month study visit retention, N (%)	255 (81)	132 (84)	123 (78)	0.240

There was no statistical difference between study arms in completing life skills (94%) or Red Cross (77%) trainings, however there was a trend towards higher completion and retention among intervention participants. Overall training completion was 82% for intervention participants, and 73% for control participants; 24 month retention was 84% for intervention participants vs. 78% for control participants.

Within the intervention arm, 124 (70%) participants initiated vocational training, however only 100 (63%) passed, and 92 (just under 60%)received a micro-grant. For the 66 participants who did not receive a start-up grant, the primary reasons included not completing vocational training and/or not developing a business plan (24);returning to formal education (11);and relocation (30) (categories not mutually exclusive). Because of loss to follow-up, it was not possible to determine either causes or consequences of migration among those who left the area during the course of the study. The microgrant opportunity should have been expected to reduce migration, but we were unable to evaluate this anticipated consequence of the SHAZ! intervention.

### Qualitative program monitoring data

Among the 24 participants who did not complete vocational training or develop a business plan, specific reasons for non-completion were difficult to assess; however programmatic monitoring data collected during guidance counselling visits revealed that participants faced language barriers, and family demands during hours that vocational training was offered. Due to the availability of training programs and the timing of completion of the required Life-skills and Red Cross components of the study, participants in the intervention arm completed their vocational training programs later than expected. Thus 78% of intervention participants had yet to fully complete intervention activities at the time of the 18 month follow up visit.

Program monitoring data also revealed that some control participants may have translated study reimbursements ($5/visit) into economic opportunities such as paying for school fees, or buying goods and selling them at a higher price.

### Effect of Intervention on Structural and Sexual Behavioural Factors

Table 2 presents results from the GEE analysis of the effects of the intervention on structural and sexual risk behaviour over the 24 month study period. Changes in outcomes from baseline through follow up within each study arm are presented with frequencies by arm across study visits, with the magnitude of change per six month period indicated by Odd Ratios and corresponding 95% confidence intervals. Tests for whether reported changes differed by study arm are indicated by the interaction p-value.

**Table 2:** Effect of the Intervention on Structural and Sexual Risk Factors.

	Baseline Visit	6 Month Visit	12 Month Visit	18 Month Visit	24 Month Visit	Within Arm	Interaction
	no./total no. (%)	no./total no. (%)	no./total no. (%)	no./total no. (%)	no./total no. (%)	OR (95% CI)	P-value
**Structural Factors**							
**Food Insecure**							
Intervention, no./total no. (%)	55/159 (35)	35/149 (23)	28/142 (20)	21/136 (15)	13/129 (10)	0.68 (0.60, 0.77)	0.02
Control, no./total no. (%)	46/155 (30)	29/143 (20)	19/137 (14)	23/118 (19)	19/119 (16)	0.83 (0.72, 0.94)	
**Received Own Income**							
Intervention, no./total no. (%)	5/153 (3)	17/148 (11)	37/142 (26)	48/136 (35)	63/129 (49)	2.05 (1.79, 2.34)	0.02
Control, no./total no. (%)	14/155 (9)	25/143 (17)	47/137 (34)	46/118 (39)	55/119 (46)	1.67 (1.48, 1.87)	
**Received High Social Support**							
Intervention, no./total no. (%)	60/158 (38)	58/149 (39)	66/142 (46)	69/136 (51)	70/132 (53)	1.18 (1.09, 1.29)	0.94
Control, no./total no. (%)	51/157 (32)	61/143 (43)	58/137 (42)	57/118 (48)	61/123 (50)	1.19 (1.09, 1.30)	
**‘High’ Relationship Power Score**							
Intervention, no./total no. (%)	29/155 (19)	36/149 (24)	52/142 (37)	42/136 (31)	53/129 (41)	1.30 (1.17, 1.44)	0.90
Control, no./total no. (%)	23/156 (15)	37/143 (26)	33/137 (24)	40/118 (34)	41/119 (34)	1.28 (1.15, 1.43)	
**Physical/Sexual Violence or Rape** *							
Intervention, no./total no. (%)	-	8/149 (5)	1/142 (0.7)	0/136 (0)	0/132 (0)	0.10 (0.02, 0.67)	0.06
Control, no./total no. (%)	-	11/143 (8)	4/137 (3)	3/118 (2)	3/123 (2)	0.63 (0.41, 0.96)	
**Sexual Behavior/Risk**							
**Had ever had sex**							
Intervention, no./total no. (%)	43/155(28)	53/149 (36)	63/142(44)	73/136(54)	81/132(61)		
Control, no./total no. (%)	36/156(23)	48/143(34)	59/137(43)	60/118(51)	70/123(57)		
**Sexually Active in Last Month**							
Intervention, no./total no. (%)	20/154 (13)	21/149 (14)	31/142 (22)	37/136 (27)	53/132 (40)	1.48 (1.32, 1.67)	0.93
Control, no./total no. (%)	17/156 (11)	25/143 (18)	32/137 (23)	41/118 (35)	45/123 (37)	1.49 (1.33, 1.66)	
**Transactional Sex in Last Month** **							
Intervention, no./total no. (%)	9/13 (69)	13/21 (62)	15/34(44)	7/33 (21)	16/48 (33)	0.64 (0.50, 0.83)	0.25
Control, no./total no. (%)	5/12 (42)	14/26 (54)	13/35(37)	16/41 (39)	12/42 (29)	0.79 (0.62, 1.02)	
**Condom Use with Current Partner** **							
Intervention, no./total no. (%)	5/14 (36)	14/16 (87)	20/22 (91)	19/21 (90)	29/33 (88)	1.79 (1.23, 2.62)	0.25
Control, no./total no. (%)	8/12 (67)	14/17 (82)	24/24 (100)	23/26 (88)	25/29 (86)	1.29 (0.86, 1.95)	
**Contraceptive Use Current Partner** **							
Intervention, no./total no. (%)	2/9 (22)	4/9 (44)	3/15(20)	6/18 (33)	9/24 (38)	1.09 (0.74, 1.54)	0.67
Control, no./total no. (%)	1/2 (50)	6/15 (40)	7/17 (41)	10/25 (40)	8/21 (38)	0.97 (0.67, 1.41)	

OR = Odds Ratio; CI = Confidence Interval.

Statistical test of difference in Odds Ratios by study arm.

*Baseline excluded because it measured lifetime experience of violence, whereas subsequent measures were based on violence experienced during previous 6-month interval.

**among those who reported sexual activity in the previous month (n = 37).

Overall, intervention participants showed a significantly greater decrease in food insecurity [IOR = 0.83 vs. COR = 0.68, p-0.02], and a higher likelihood of receiving their own income [IOR = 2.05 vs. COR = 1.67, p = 0.02] overtime, compared to control participants. In addition, intervention participants had a greater reduction in the experience of violence overtime, of marginal statistical significance (although overall prevalence for this outcome was very low) [IOR = 0.10 vs. COR = 0.63, p = 0.06].

Participants in both arms initiated sexual activity, an expected outcome given the aging of the cohort over 24 months during a period when adolescents are likely to become sexually active. Overall, 59% of the respondents had initiated sexual activity by the end of the study, compared to 25% at the beginning of the study. There was no difference in the proportion who experienced sexual debut by study arm. However, among participants in the intervention arm there were statistically significant changes over time in the risk of transactional sex [IOR = 0.64, 95% CI (0.50, 0.83)], and likelihood of using a condom with their current partner [IOR = 1.79, 95% CI (1.23, 2.62)] from baseline through study follow up.

Over the study period, participants reported statistically significant increases in social support relationship power, and sexual activity that were virtually the same across study arms (See Table 2 for details). No statistically significant changes were reported for contraceptive use (other than condoms as described above) in either arm.

### Effect of the Intervention on Biological Outcomes

Overall incidence was very high at: 2.3/100 women years (wy) for HIV; 4.7/100 wy for HSV-2; and 10.8/100 wy for unintended pregnancy (Table 3). The study was not powered to detect differences in biological outcomes, and hazard ratios for HIV and HSV-2 were not statistically significant. HIV incidence was the same between the two groups [HR = 0.94, 95% CI (0.33, 2.69)], while HSV-2 incidence was slightly higher in the intervention compared to the control group [HSV-2 HR = 1.50 (0.70, 3.19)]. However, the study did show a borderline statistically significant reduction of 40% in unintended pregnancy among intervention participants compared to control participants[AHR = 0.61, 95% CI (0.37, 1.01)], suggesting a trend towards greater fertility control among those in the intervention arm. Accounting for informative censoring using inverse probability weighting did not change these results.

**Table 3:** Effect of participation in the intervention arm (vs. control) on biological outcomes.

	No./Total No.	Person-years of Follow-up	HR (95% CI)	P-value	HR IPCW(95% CI)
**HIV**					
Intervention	7/158	309	0.94 (0.33, 2.69)	0.913	1.02(0.35, 2.96)
Control	7/157	295	- (Ref)		
**HSV-2**					
Intervention	17/158	299	1.50 (0.70, 3.19)	0.298	1.60 (0.74, 3.49)
Control	11/157	292	- (Ref)		
**Unintended pregnancy**					
Intervention	26/158	303	0.62 (0.38, 1.02)	0.061	0.61 (0.37, 1.01)
Control	37/157	279	- (Ref)		

## Discussion

The results of this Pilot Intervention evaluation showed that we were able to identify, recruit and retain over two years a large number of adolescent women for study participation, in spite of a challenging economic and political context. The evaluation revealed promising effects of intervention participation on structural and behavioural outcomes, such as significant improvements and/or expected trends among intervention participants in improving economic factors (food security and income) and decreasing HIV risk factors (e.g. transactional sex, condom use and experience of violence). While we did not observe expected trends between study arms in HIV or HSV-2 incidence, it is important to note that we saw an impressive 40% reduction in unintended pregnancy of borderline statistical significance despite not being powered for that outcome. Such results suggest important potential for the SHAZ! model to mitigate HIV risk among out-of-school adolescent female orphans, even in challenging economic contexts, and echo findings from the IMAGE study as reported in the introduction. Findings are also consistent with preliminary data from a recent “Stepping Stones/Creating Futures” evaluation that assessed life skills and livelihoods training among 233, 18–24 years olds, both men and women, in Durban, South Africa. This evaluation showed a 50% increase in income earned by women at three months post intervention, and a decrease in reports of transactional sex (from 72% to 55%) [54].

In addition, on many of the outcomes studied, a review of “pre-post” indicators (across all study participants, not stratified by study arm)reveal highly statistically significant improvements for critical social factors (e.g. social support received, relationship power) that were not statistically different across arms. There are a few possible explanations for why this might have been the case. First, given the individual RCT design, it was difficult to define a true “standard of care” control arm. Because the study team felt ethically obligated to offer access to the same HIV and SRH education and services, and due to the fact that we followed up control participants who did not show up for study visits, the control condition received far more care and support than they would have had they not been enrolled in the study. In other words the control condition likely provided more benefit than a true control condition or standard of care would have. These factors would have underestimated the full effect of the intervention and/or reduced our ability to detect the marginal intervention effects of the combined intervention. It is also possible that the evaluation period or the length of the intervention was too short to capture the hypothesized benefits of the livelihoods intervention over and above the control condition on longer term behavioral or health outcomes. Because only 22% of intervention arm participants were able to fully complete vocational training prior to the 18 month visit, the benefits of economic activity related to that training may not have been realized until after the final study visit. Furthermore, participants and study staff could not be blinded to which study group was assigned, which may have impacted participation, behaviors and assessment of outcomes. However, ACASI was used to collect most of the behavioral and economic outcome data, and ensured that the collection of data was not biased based on study arm assignment. There may also have been some “contamination” across arms where control participants converted study reimbursements ($5/study visit) into economic opportunities - such as paying for school fees or buying and selling goods at a higher price. Although we did not capture this activity in a systematic way, program monitoring data indicate this did occur among some participants. A detailed analysis of qualitative data collected through in-depth interviews with a sub-sample of study participants is underway and may shed light on some of these remaining questions.

Another limitation when researching sexual behaviour among adolescents in longitudinal studies is related to sexual behaviour and age effects. While our population range was 16–19, the mean age of participants at baseline was 18 years, corresponding to the mean age of sexual debut for women in Zimbabwe [55].We therefore expected that sexual activity in our study would likely increase over the two year period; however we hoped that is would remain steady or increase less among intervention participants. While we did not see any difference between study arms, it is important to note that all study participants reached the age of 18 by the end of follow up, however only 59% had initiated sexual activity and almost all who had initiated sexual activity(92%) reported only one sexual partner during the course of the study. In the Zimbabwe Demographic and Health survey conducted in 2005/2006 (close to the time period when SHAZ! was rolled out) reported initiation of sexual activity increased from 32.1% among 15 to 19 year olds to 83.5% among women 20 to 24, and the age of sexual debut was lower for those with less education and greater poverty [56].This suggests that participants in both the SHAZ! Pilot Intervention and control arms were no more likely to initiate sexual activity than the general population, and may have had reduced risk for sexual initiation by participating in study.

Finally, the environment in Zimbabwe (particularly the hyper-inflation) at the time made it difficult to test “proof of concept”, and, as mentioned, only 60% of the participants ultimately received a micro-grant due, in part to the overarching context, and to difficulties in completing vocational training courses and developing business plans. While this was an important finding in itself for ongoing program improvement, such challenges rendered it difficult to determine the full effect of the economic intervention. It is important to note that in-spite of the overarching economic challenges, however, intervention participants did experience improvements in economic factors, suggesting that the SHAZ! Pilot Intervention had an impact even in a highly challenging setting. Ongoing analysis of qualitative data, not yet fully analyzed, will hopefully be of use in interpreting the findings presented in this paper.

In spite of these challenges, the study had several strengths, primarily the rigorous RCT design, the community-based process to design the intervention, and the use of ACASI and “young” face-to-face interviewers to improve the reporting of sensitive behaviors, and objective measures of biological outcomes. In addition, retention was higher than expected given the political and economic turmoil during the period, and the chaotic lives of adolescent orphans who are often shuttled between extended family households. This seems to reflect strong motivation among participants, and effective retention and outreach efforts by the study team. Adjusting the analyses for censoring did not change the findings of the study.

Given the urgent need to identify strategies that effectively reduce HIV risk among adolescent women in sub-Saharan Africa, it is critical that researchers and program staff take the lessons-learnt from the SHAZ! Pilot Intervention and apply them to ongoing and future programs and evaluations. Such lessons include, for example, that any future fielding or testing of the model should incorporate a wider-range of livelihoods options in the local language from basic short courses to more advanced training opportunities, with flexible hours to better meet the varying needs of adolescent women. These changes would help ensure that a greater proportion of participants could take full advantage of the economic opportunities provided. In addition, we recommend that future studies be implemented in a range of setting, including more stable economic and political environments than the one provided by Zimbabwe during the study period, to assess the broad potential of the model to be effective through the sub-Saharan African region. The extreme economic crisis in Zimbabwe at the time hindered the ability to fully evaluate the model and limits generalizability to more stable contexts. Finally, we recommend that any future evaluations consider other experimental and quasi-experimental designs that allow comparisons among amore true standard of care controls. For example, evaluators could consider phased roll-outs at a community level, where intervention communities are compared to those that are yet to receive the intervention. This would allow the research community to more clearly assess the effect of the intervention compared to standard of care.

This study reflects the challenges inherent in rigorously evaluating structural interventions among most-at-risk populations, where they are needed most. Through this evaluation, SHAZ! emerges as a promising model to build on towards preventing HIV among and improving the overall health and well-being of adolescent women. Ongoing work to address the inequitable burden of HIV among adolescent and young women in Africa, incorporating the lessons-learned from this study, is of critical importance.

## Supporting Information

Checklist S1 **CONSORT Checklist.**
(DOC)

Protocol S1 **Trial Protocol.**
(DOC)
