# Supplementary material for: The SHAZ! Project: Results from a Pilot Randomized Trial of a Structural Intervention to Prevent HIV among Adolescent Women in Zimbabwe
Source: PLoS One. 2014 Nov 21;9(11):e113621. doi: 10.1371/journal.pone.0113621 (PMC4240618; doi:10.1371/journal.pone.0113621)
Supplement: Protocol S1 — Trial Protocol. (DOC) [file pone.0113621.s002.doc]

**Economic Opportunity for Zimbabwean Adolescent Orphans**

1. **Study Aims**

The overall aim of this project is to determine, using a randomized controlled design, whether a economic livelihood (in this case vocational training package including monthly fees, a modest stipend based on progression and performance in the training program, and a completion bonus) and life-skills intervention, designed to increase control over sexual interactions, prevents the adverse consequences of unprotected sex among out-of-school female orphans aged 16-19 in Zimbabwe compared with a life-skills intervention alone.

**Aim 1: To assess the effectiveness of a combined vocational training package and life-skills education intervention in reducing the incidence of our primary outcomes: Herpes simplex virus type 2 (HSV2) and unintended pregnancy.** Although not powered to assess HIV as an outcome, we will nevertheless monitor HIV incidence between groups. (Note that rates of bacterial STDs are too low to consider.) We hypothesize that compared to those in the control group, adolescent female orphans receiving the combined intervention will experience a lower incidence of these outcomes.

Aim 2: To assess the effectiveness of a combined vocational training package and life-skills education intervention in reducing our secondary outcomes: high-risk behaviors (e.g., unprotected sex, number of partners, transactional sex, early sexual debut, sexual coercion and violence). Our hypothesis is identical to that for Aim 1.

**Aim 3. To examine the intermediary effects of increased economic opportunity, social support, and improved sexual negotiation skills in enhancing participants’ control over sexual interactions.** We hypothesize that the combined intervention will enable participants to, for example, increase and gain control over their income and future income-earning potential (through the vocational training intervention), thereby empowering them to use the sexual negotiating skills gained through the life-skills intervention. Together, these outcomes will increase participants’ control over sexual activity, and reduce the prevalence of sexual risk behaviors and the incidence of both HSV-2 and unintended pregnancies. This aim will allow us to examine why the intervention is or is not effective. Furthermore, if the predictive value of these outcomes is high, this may obviate the need for collection of biological data and self-reports of sensitive behaviors in future studies.

**Aim 4:** **To evaluate whether characteristics related to participation, i.e., factors related to participant adherence, satisfaction, and understanding of the intervention mediate observed effects.** We will monitor participants’ adherence to the intervention (e.g., attendance at life skills and vocational trainings, required meetings), level of satisfaction with the program, and understanding of intervention content. We hypothesize that greater adherence, satisfaction, and level of understanding will be associated with positive biological and behavioral outcomes.

**Aim 5: To test the feasibility of expanding the intervention as designed to HIV and HSV-2 positive participants, and to test the feasibility of the intervention’s potential to support positive participants. (referred to as the *Prevention for Positives* component)** We hypothesize that with minor adjustments the intervention as designed will be adequate to meet the needs of positive clients in a fully integrative and confidential manner, with minimal fears of disclosure and without resultant discrimination or stigma. We hypothesize that the combined behavioral and economic intervention (intervention arm) will be more effective in empowering positive adolescents with improved economic livelihoods, better access to care and treatment, and slower disease progression, and will also effect behavior to prevent co-infection with other STI's and secondary transmission to partners, compared to the life skills intervention alone (control arm). We will monitor and record issues that arise through observation, in-depth interviews, supplemental questionnaires and trainer reports and to use these results to further refine the integrated social support facets of the intervention for future scale-up.

**2. Background**

**A. HIV/AIDS, HSV-2, and high-risk behaviors among adolescent females in Zimbabwe**

Zimbabwe has one of the highest HIV infection rates worldwide, at an estimated 30% [6]. Zimbabwe’s HIV epidemic has a disproportionate impact on adolescent girls. Approximately 25% of Zimbabwean females aged 15-24 are infected with HIV, compared to 11% of like-aged males [8]. HSV-2 prevalence levels of up to 45% have been found among females aged 15-19, making it the most prevalent STI among Zimbabwean women under 20 years of age [9, 10]. Fifteen percent of girls in Zimbabwe have begun childbearing by age 19 [12].

B. HSV-2 as an indicator of sexual risk behavior and relevance to HIV transmission

The prevalence of genital herpes virus, type 2 (HSV-2) infection as measured by antibody to HSV-2 is considered an excellent objective indicator of sexual risk behaviors [5]. Serologic surveys of HSV-2 antibody have shown that persons with high-risk profiles, such as STD patients and female sex workers, have very high HSV-2 prevalence; and persons with low risk profiles have low or zero HSV-2 prevalence [5]. In addition, HSV-2 is considered the most important facilitating factor for HIV transmission in developing countries [13-15] most likely because HSV-2 is the major STI associated with genital lesions, which are known to increase the transmissibility of HIV [9, 10, 16-19].

**C. AIDS orphans**

The AIDS epidemic, in tandem with Zimbabwe’s economic crisis, has contributed to a dramatic increase in the number of orphans. According to the 1999 Zimbabwe Demographic and Health Survey, less than half of children under 15 years of age (46%) are living with both parents. Adolescents without parents are far less likely than other adolescents to have access to educational and economic opportunities. They are also often stigmatized, vulnerable to poverty and thus malnutrition, illiteracy, and unemployment, all of which can lead to exploitation, sexual abuse and HIV infection, [22] [23].

**D. Economic decline in Zimbabwe and its effects on adolescent female orphans**

Zimbabwe is currently undergoing an economic crisis with inflation at over 144%, and unemployment at 65%; most Zimbabweans earn less than US$550 per year [13]. Zimbabwe’s economy is shrinking, with a –12% growth rate in Gross Domestic Product. This has lead to a dramatic increase in the number of Zimbabwean’s living in poverty, with a current 80% of the population living below the poverty line [13]. The rapidly declining economy can no longer support sufficient agricultural production, which has lead to growing food shortages [24]. Adolescent females, especially orphans, have few economic opportunities or options to mitigate the effects of poverty. Therefore, many orphan girls are struggling to support themselves and often dependent siblings, and are at high-risk for engaging in transactional sex for survival.

**E. Transactional sex**

Data on adolescent sexuality and reproductive health from across Africa, Asia, and Latin America uniformly indicate that HIV risk behaviors are intimately connected to the social and economic context of young people’s lives [27]. Economic hardship is associated with sexual risk behaviors in young women, including multiple partnerships, older partners, and transactional sex, a well-documented phenomenon in Sub Saharan Africa [28-39]. A recent survey in Zimbabwe found that 13% of all unmarried females aged 15-19 recently received money/gifts in exchange for sex [40]a problem that is estimated to be much more prevalent among adolescent orphans [41].

###### F. Economic empowerment through livelihood development programs and reproductive health

###### In recognition that economic vulnerability is associated with HIV risk, and burgeoning numbers of AIDS orphans and vulnerable youth, there has been increasing interest in ‘livelihood development’ (eg. microcredit) interventions for at-risk youth, particularly among girls. ‘Livelihoods’ comprises ‘the capabilities, assets and activities required for a means of living’. Research shows that micro-credit among females in sub-Saharan Africa may have potential to decrease HIV rates [44, 45]. A large and varied number of adolescent livelihood interventions have been attempted. Many of these interventions have combined economic livelihood development with reproductive/sexual health education components. To date however, few studies of economic livelihood programs have looked specifically at reproductive health outcomes and no rigorous evaluations have examined the effect of such programs on biological outcomes [70, 87-90,]. The major hypothesis is that economic options allow women a stronger decision-making role in reproductive behavior, with commensurate effects on health outcomes.

**G. Life-skills Education and Reproductive Health**

Life-skills Education programs, also called Skills-Based Health Education, have been used for a variety of education and prevention programs, particularly sexual and reproductive health including training in sexual negotiation skills. These programs go beyond the traditional didactic information sessions, by adopting gender-sensitive, interactive, participatory methods that not only inform, but also help change attitudes and behaviors[53]. A review by UNICEF found that educational efforts with a life-skills approach were effective in behavior change. Stepping Stones, one of the curricula upon which the SHAZ! lifeskills training is based, has been implemented in a variety of cultural settings and age groups, including adolescents in sub-Saharan Africa [54]. The UNICEF report found that the Stepping Stones Program resulted in a decreased number of sexual partners, and more use of condoms among attendees [55]. That said, these evaluations occurred immediately after program implementation and there are no data on sustained change. In fact, researchers have concluded that only providing HIV/reproductive health education is a first, but inadequate, approach to sustainable behavior change [57, 58].

**H. SHAZ! Pilot Intervention Study**

From March to December 2004, we tested the feasibility of a combined economic and life skills intervention among 16-19 year old female orphans and the potential for it to address HIV/STI risk and enhance reproductive health. The project, entitled SHAZ! (Shaping the Health of Adolescents in Zimbabwe) was carried out in Epworth and Chitungwiza, peri-urban and high-density urban areas respectively, located on the outskirts of Harare, from March to December 2004 (CHR # H6070-21521-02A, MRCZ/A/983). The intervention combined life skills education, business training, mentoring and the provision of micro-credit.

**1. Intervention Overview**

Participants received on average 15 weeks of training in business, mentoring and life skills education. The culmination of the training resulted in the development of a business plan that was assessed by a micro-credit partner. If deemed viable, participants were provided with a loan (ranging from US $51 to $87) to start-up their proposed economic project. Using a group-lending model, loans and repayment were provided through peer support groups, during which ongoing life skills education was also supported. Each participant had access to a business mentor to turn to for assistance and support in running businesses. Participants were followed up for 6 months.

**2. Pilot Study Design**

We conducted a cross-sectional study among 200 in and out of school girls between 16 and 19 years of age living in our research sites. Convenience sampling was used to recruit participants into the study. Participants were enrolled at clinic locations in both sites, as well as through a mobile clinic facility. A subset of 50 orphans (defined as having lost at least one parent) was enrolled into the pilot intervention and was followed up at 3 and 6months.

Information at baseline and follow-up visits was collected from study participants using both quantitative and qualitative methods of data. Quantitative surveys, using face to face interviews and ACASI, were conducted at baseline, and at 3 and 6 months to collect information on demographics, HIV and RH knowledge, sexual behavior and contraceptive use, partners, relationship power and current economic activities. Biological specimens for HIV, HSV-2 and pregnancy were collected and tested. Qualitative data was collected through observation and in-depth interviews throughout the pilot to provide information on implementation quality and to assess successes and failures of the intervention. Process evaluation data on training attendance, loan disbursement, loan repayment, and business assessments were also collected. The main outcome variables assessed were socio-demographic characteristics, sexual behavior, control over sexual interactions, sexual and physical abuse, participation in and knowledge gained from training, micro-enterprise activities, including income generation, and HIV, HSV-2, and pregnancy status.

3. Findings

The pilot study cross sectional survey (n=200) showed that orphans and out of school girls were more likely to be sexually active (40% vs 24%), have a higher prevalence of HIV (9% vs 3%), HSV-2 (10% vs 6%) and to have had a past or current pregnancy (15% vs 5%). Among the 50 enrolled in the pilot intervention, 43% had ever had sex, 57% and 14% had ever used male and female condoms respectively and 14% had ever been raped. Fifty seven percent had been sexually active in the last three months and 44% received basic needs/money from primary sexual partner at baseline.

By the end of the study, attendance at business and life skills training sessions averaged 80%, and 80% of participants developed business plans and received loans. Business activities included buying and selling (maize, foodstuffs, household items, clothing), home décor, sewing and hairdressing. While the percentage of participants reporting having their own income increased from 6 to 44% (p<0.01), only 5% had made repayments on their loans. Qualitative research indicated that those who did succeed had wide family and/or other social support in running their businesses, including participating in family owned businesses. While the study was not powered to detect differences between baseline and follow-up outcomes, statistically significant findings were found in knowledge and relationship power. The percentage of participants that answered all HIV questions correctly increased from 16% to 38% (p<0.001), and achieving a high relationship power score increased from 11% to 50% in primary sexual relationships (p=.02) and from 5% to 8% in non-sexual relationships (p=.07). Relationship power was associated with condom use at baseline: 80% of those with high relationship power had ever used condoms compared to 44% with low relationship power.

In sum, adolescents in the study faced challenges in utilizing micro-credit, but pilot results indicated that the combined intervention did have the potential to reduce risk through increasing knowledge and relationship power. Social support is a key to success in economic endeavors.

**3. Significance**

**The need for a rigorous evaluation of an economic livelihood and reproductive health education intervention**

Although there is great potential for economic livelihood programs to improve the reproductive health of young women, including adolescent female orphans, no research to date has assessed the effect of economic interventions on sexual risk behaviors and STI/HIV and pregnancy outcomes. Furthermore, no studies have examined the intermediary effects of such interventions (e.g. increased income) on enabling participants to control their sexual activities and thus decrease risky sexual behaviors and thereby reduce adverse biological outcomes. An understanding of the effects of such programs and the process by which economic factors affect adolescents’ sexual risk behaviors, and risk of STIs/HIV and unintended pregnancy is critical to the design and promotion of effective interventions. Our pilot work suggests that micro-credit as a main economic livelihood intervention for adolescent female orphans in this setting is not appropriate, but that the a combined economic livelihood and behavioral intervention has potential to reduce risk, as long as it includes extensive training and enhanced social support.

**4. Methods**

**A. General Study Design**

We propose a randomized controlled trial of an intervention combining a vocational training package -- including a conditional stipend and completion bonus -- with life-skills education and social support, compared to life-skills education alone. The study will begin with six months of start-up activities, and enrollment will take place within a six-month period at the beginning of the study. The economic livelihood intervention and life-skills programs will be administered for one year and participants will be followed for one year post-intervention. Data collection and service provision to participants will be provided through a health clinic and occasional visits using a mobile clinic. The frequency of intervention visits (i.e. visits related to the intervention program such as life-skills training, SHAZ reunions, and vocational training) is described below. Visits for data collection, including biological and behavioral outcome data, will be collected at baseline, and every six months thereafter for a total of five visits over the three year period. We will follow participants for one year after the intervention to ensure sufficient power to examine our primary outcome (Aim 1) as well as to establish the temporal association between the intermediary effects of increased economic opportunity and improved sexual negotiation skills in enhancing participants’ control over sexual interactions (Aim 3). Finally one year of follow-up will allow us to assess the intervention’s sustained effects on high-risk behavior (Aim 2). (Please see appendices: Intervention Design and Study Design Flow Chart)

**Prevention for Positives component:** The SHAZ main study has completed its enrollment for the ongoing intervention study, with a total of n=315. In order to assess the feasibility of implementing this intervention with HIV and HSV-2 positive clients, we will enroll an additional cohort of n=50 according to the same criteria as was used in the main study, except that those who are HIV and/or HSV-2 positive also will be eligible to enroll.

In order to meet this objective, we will contact and invite all participants who screened out of the study because of positive results (n=40) and invite these to re-screen for the study. We will then continue to recruit additional participants until we meet the desired sample size n=50.

We will randomize participants into intervention and control arms, as per the main study, and deliver the intervention, study visits and data collection activities according to existing SHAZ protocols**. The exception is that the Prevention for Positives cohort will have only two follow up visits instead of three.** The participants of this feasibility study will then participate in the intervention just as is described in detail in the original main study protocol.

#### Methods of Data Analysis

**1.a. Sample Size – Quantitative**

Sample size calculations for Aim 1 assume that the background annual incidence rate for HSV-2 is 16% (corresponding to a prevalence rate following two years of follow-up of approximately 22%), and that participants will be followed for three years. Further, we conservatively assume that 20% of participants will be lost to follow-up (our average loss to follow-up over all completed studies is 12% [C.1]). Although individuals will form the unit of randomization, the fact that they will be associated via membership in life skills cohorts may induce correlation between individual outcomes within groups (assumed to include an average number of 25 girls) –We account for this using methods described in Hayes and Bennett [77], which take into account both the number of individuals randomized into each arm and the between-group variation in incidence rates induced by within-group correlation in infection outcomes. Incidence rates will be compared between arms using a two-sample t statistic adjusted to account for this source of variation. The corresponding coefficient of variation is assumed to be 0.2, a conservative estimate similar to values used in other studies [77, 78]. With these assumptions, our proposed sample size **of 300 (150** individuals randomized to each arm) will provide 80% power to detect a relative reduction in HSV-2 incidence as small as .53 at the 5% significance level. This corresponds to a 47% reduction in HSV-2 incidence (from 12% to 6.3%). Because unintended pregnancy is expected to have similar, if not higher incidence rates, the above calculations apply to this outcome as well.

Similar calculations for the HIV infection outcome (at 8% prevalence) indicate that we should have 80% power to detect a relative risk comparing incidence in the intervention group to the control group as small as .29 at the 5% significance level. This corresponds to a 70% absolute reduction in HIV incidence, assuming a background annual HIV incidence rate in the target communities of approximately 4%. This is an effect we do not expect to be able to detect given our sample size. We will, however, be able to report trends. The high prevalence rates cited in section B.1. were among young females in an antenatal clinic who were obviously sexually active and practicing unprotected sex. Among adolescents in Zimbabwe, HSV-2 rates tend to be three to five time higher than HIV rates [10].

Additional calculations for Aim 2 are based on comparing observed changes in self-reported behaviors between arms using a t-test with adjustment for within-group correlations as described above. For these calculations, we assume changes will be computed as binary indicators of decrease in risk-taking behavior between the initial and final visits (e.g., a decrease in self-reported unprotected sex, or in the annual number of sexual partners). We also assume that the background prevalence of individuals reporting a decrease in risk-taking behavior ranges between 10% and 50%, and that the between-cluster coefficient of variation in prevalence within arms is 0.2. With these assumptions, our planned sample size should provide 80% power to detect prevalence ratios comparing prevalence of behavior change in the combined intervention group to that in the control group in the range .55-.7 at the 5% significance level. This corresponds to absolute differences in prevalence between arms of .07 – x.15, indicating that we should have sufficient power to detect relatively small differences between groups for a range of observed changes in high-risk behaviors.

Formal sample size calculations are not presented for Aims 3 or 4 because detailed preliminary information on the joint distribution of behavioral (including characteristics of participation) and our primary and secondary outcomes do not exist, and because the analyses proposed for these aims (described in section D.11.) are exploratory in nature. Since these analyses may involve estimation of interactions, we will clearly have reduced power compared to the analyses for Aims 1 and 2. However, we anticipate that power will be adequate to detect important associations.

1.b. Sample Size – Qualitative Component

Adolescent Girl Participants: Forty main study participants (20 from the intervention arm and 20 from the control arm) will be selected for participation in the qualitative interviews. As participants are enrolled into either study arm, every 5^th^ girl in both arms will be selected for participation. The 40 participants will be interviewed 3 times for the duration of the study for a total of 120 interviews.

Interviews from an additional 10 participants enrolled in the Prevention for Positives component (5 from each arm, randomly chosen from the new cohort of 50) will be added to explore the feasibility of the study to include and support HIV and HSV-2 positive participants.

Household Heads: For each participant invited to undergo an in-depth interview, researchers will also interview their household head if the household head agrees to an interview. The 40 household heads will be interviewed one time only during the study for a total of 40 interviews.

Community and Youth Forum Members and Community Leaders: Four Community Advisory Board members (CAB) and two Youth Forum (YF) members will be interviewed every eight months for duration of the study; 3 interviews/participant providing a total of 24 interviews. One-time interviews will carried with identified community leaders (including religious leaders) or other key adult residents of Chitungwiza; the total number is not yet determined. Two key persons or trainers from each of the training institutions (3 identified institutions) working in partnership with the SHAZ! Study will be interviewed at three different times for the duration of the study providing a total of 18 interviews.

Adult Men: 30 adult men will be randomly selected for in-depth interviews. Men will be recruited from three identified locations where Zimbabwean men socialize and relax: beerhalls, sports clubs and at local “braais” or barbeques where informal sports games are often also played. Counting every fifth person who arrives ten men/location will be recruited. Interviewers will then secure a private time and place where in-depth interviews can be held.

2. Data Analysis

The primary analysis for Aim 1 will be based on an intent-to-treat comparison of HSV-2 incidence across treatment arms using Poisson regression methods to account for duration of follow-up. Individuals (the primary unit of randomization) will be the basic unit of analysis in these comparisons. However, we will control for correlation in incidence between peer support groups using generalized estimating equation (GEE) regression methods [85] appropriate for clustered outcomes. A similar approach will be taken to compare rates of unintended pregnancy and HIV infection across arms.

The effect of the combined intervention on reduction in self-reported risk behaviors (Aim 2) will be evaluated by comparison of the difference in average behavior change between arms. Changes will initially be computed as binary indicators of decrease in behavior between the initial and final visit, and results assessed via the between-arm difference in proportions. Similar to the approach described above for Aim 1, GEE methods will be used to account for potential correlations between outcomes within peer groups. We will compare absolute prevalence of risk behaviors at successive follow-up visits in a similar fashion.

The goal of Aim 3 will be to investigate the association between intermediary effects of the proximal outcomes of intervention and sexual negotiation skills as well as control over sexual interactions, in an effort to understand possible causal links. The analyses will focus on girls in the intervention arm. Intermediary outcomes will be quantified using a variety of measures, including successful completion of vocational training, utilization of social support /counseling, control over economic resources and decision making, self-efficacy and risk-related behavior (using economic modeling), future goals and aspirations.

Analyses of the relationship between characteristics of participation: adherence, satisfaction and understanding, to biological and behavioral outcomes (Aim 4) will be similar to those presented for Aim 3 and will focus on participants in the intervention arm. For example, adherence will be measured using visit-specific categorical and continuous summaries of participation in program activities (e.g. attendance at training sessions). These will be related as time-varying covariates to primary (biological) outcomes using discrete time survival analysis techniques as described for Aim 3. The approach for secondary (behavioral) outcomes will also be based on visit-specific indictors of current behavior, which will be related to adherence using GEE regression methods for binary outcomes. As before, we will control for membership in peer groups in the analyses.

For the qualitative component, data will be transcribed from audio tapes and translated, then entered into Atlas.ti for qualitative data analysis. The transcripts will be coded and themes identified that will be tabulated to help inform all aspects of the data analysis process.

**C. Subject Selection**

i. Main Study

**1. Who and Why**: The target population for the study is out-of-school adolescent female orphans living in urban -Zimbabwe. Females aged 16 to 19 years will be recruited. We restricted the intervention to females because prevalence data clearly show that girls are at higher risk for HIV infection. We hypothesize that an economic livelihood intervention will have greater effect on our biological and behavioral outcomes in females, as it will create alternatives to transactional sex, which is overwhelmingly practiced by females for accessing resources and gaining status. Sixteen is the legal age of consent in Zimbabwe, thus, we are able to collect and test biological specimens and provide results without obtaining parental or /guardian consent. We have chosen our upper age range as 19 because this is consistent with literature on the definition of adolescents [69]. We have restricted our sample to those girls not currently in school, and who have not already graduated from secondary school. The vulnerability of out of school girls (reflected through current HIV trends) also indicates a lack of access to: knowledge of health risks, skills in communication and negotiation, and to condoms and youth friendly services, which provide needed services at a convenient time and place, in a manner that satisfies adolescents’ concern over confidentiality [70, 71].

**2. Numbers**: One hundred fifty individuals will be randomly assigned to the treatment arm and 150 to the control arm, for a total of 300 participants.

**3. Inclusion/Exclusion Criteria:**

i. In order to be eligible for the study, a participant must be female; an orphan (defined as having lost at least one parent); aged 16-19; out-of-school; willing to undergo HSV-2, HIV and pregnancy testing; HSV-2 and HIV negative; not pregnant; able and willing to sign an informed consent form to join the study; willing to be randomized and, if selected for the control group, to defer receipt of the economic livelihood aspect of the intervention until after the study is complete (assuming the intervention is successful); and able to speak Shona or English.

ii. Prevention for Positives cohort: An additional cohort of 50 participants will be recruited from the same population as the main study to test the feasibility of a expanding the study to HSV-2 and HIV positive participants and explore the process of integration and enhancements necessary for future scale up. We hypothesize that the inclusion of positives into this study will, with minor adjustments, be more effective in empowering positive adolescents with improved livelihoods, better access to care and treatment and slower disease progression (compared to LifeSkills alone). Additionally, we hypothesize that positive girls will not experience additional marginalization and stigma within the study or community as a result of their disease status. The only difference in inclusion/exclusion criteria for the additional 50 girls is that those who test positive for HSV-2 or HIV will not be excluded from the study

**D. Subject Recruitment – Main Study**

**1. Sources:** In Zimbabwe, orphans and their families are eligible for funds collected through a countrywide AIDS tax paid by private citizens and businesses***.*** To distribute the funds, which are delivered on a monthly basis, each District AIDS Action Committee (DAAC) has developed and maintains a list of orphans, their ages and addresses through annual door-to-door and street-based enumeration. The DAAC under which Chitungwiza falls has agreed to assist us in recruiting orphans by referring them from their lists, while maintaining the confidentiality of the potential participants We will also be recruiting participants from community based venues such as market places and youth halls.

**2. Initial contact method:** During their monthly visits, DAAC members will describe the study to potential participants and invite those interested to contact us via visits to the clinics, email, telephone or project offices. When potential participants contact us, study staff will provide additional information about the study and will determine their eligibility. Participants recruited by study staff members will similarly be invited to visit the clinics and be screened for eligibility.

3. **Prevention for Positives cohort**: Approximately 40 participants screened for the main study were positive for HIV and/or HSV-2 and therefore not eligible. We will contact these 40 participants and invite them to re-screen for the study. If they are still eligible for the study for all other criteria, they will be invited to enroll. We will continue to recruit participants using procedures already described and approved (D, 1 and 2 above) until the desired sample size of 50 is reached. In this way stigma associated with being part of a new “positive” cohort will be avoided as the additional participants enrolled to meet our sample size will include both positive and negatives. No language or recruitment methods (flyers, outreach) will be employed that imply the new cohort is a “positive cohort’, nor single out any participant as positive throughout the process, and no changes will be made to recruitment documents. Additionally, the Chitunguiza CAB who is very supportive of Shaz, will be kept updated and involved from the beginning and will help manage any rumour or related misconceptions.

**ii. Qualitative Component**

1. Who and Why: In addition to the subset of 40 adolescent girls, the qualitative component will also interview community and youth forum members and community leaders; household heads and adult men. We hypothesize that interviews with these selected participants will help broaden our understanding of what girls may be experiencing in their communities and in the intervention by discussing issues with community member, their adult care-givers and adult men.

2. Numbers: a) **50** adolescent girls, b) 40 household heads, c) 4 CAB members, d) 2 youth forum members, e) 9 community leaders and f) 30 of men will be selected for interviews.

3. Inclusion/Exclusion Criteria: In order to be eligible for the study, a participant must be a) a study participant; b) the household head of a selected participant; c) an institutional leader d) a CAB or youth forum member for at least 6 months; e) an active community leader, or f) a man aged 25 years or older, living and working in the greater Harare area. All participants, except men who will be recruited in specific male-centered locations, must be residents of Chitungwiza and able to speak Shona or English. 4. Subject Recruitment –Qualitative Component

Sources: Participants for the qualitative component will be selected through their participation in the study or through their membership within the CAB or Youth Forum. Community leaders will be identified by study participants, CAB members and/or youth forum members. Men will be recruited from three designated sites where men congregate for informal social activities: beerhalls, spots clubs and “braais” or barbeques which often include sports.

**E. Consent Process and Documentation**

All potential study participants will have the opportunity to meet with study staff one-on-one to learn about the study and ask questions for clarification. The participants will be told that they are being asked to participate in a confidential study about improving economic opportunities and reproductive health among adolescent female orphans. They will be told that participation involves periodic interviews and biological sample collection for HIV, HSV-2 and pregnancy testing, whether or not the participant is sexually active. Randomization will also be explained and participants will be told that if they participate, and if they are selected for the control group, they will receive the economic component of the intervention after the study is complete, assuming we observe an efficacious result. They will be told that some of the questions examine high-risk sexual behaviors, as well as other sensitive issues. They will be told that trained nurse/counselors will collect biological specimens and administer data collection surveys every six months for a period of three years. Participants will be told that they have the right to refuse or withdraw from the study at any time. The research objectives, the tests performed, and the names and telephone numbers of the interviewer and local principal investigator/project director will be told verbally to all participants, and summarized in an “information sheet,” with which they will be provided. If the individual is eligible and agrees to participate, study staff will explain the terms of the study’s consent form. If the prospective participant consents, she will be asked to sign the consent form. The consent form will be available in Shona and English. It may be intimidating for young people to sign a consent form, and they may feel that it will impinge upon confidentiality. Thus, participants who feel uncomfortable signing the consent form will be asked to put a mark (but no signature) at the bottom of the document to denote their consent.

Participants will be asked to take a letter home to their selected guardian that the guardian will sign in support of the participants. This, however, will not be required of any participant but will help foster guardian support for participant involvement. A subset of guardian homes will be visited by study outreach staff (with participant permission) to allow comparison of guardian interest/response rates to sending letters home with participants. At no time will study staff divulge to anyone else, including parents or guardians, any personal health information about the participant without written consent.

**F. Procedures**

**1. Study Procedures**

A. See Appendices 1 and 2. As described above, The District AIDS Action Committees (DAACs) in Zimbabwe will refer potential participants to our study, and study staff will recruit from public venues. If an individual is interested in participating, and consents to the study, she will sign the consent form and undergo a short self-administered screening questionnaire to determine eligibility. She will also undergo pre-test counseling for HSV-2, HIV and pregnancy, conducted in private at the family health or mobile clinics, and provide blood spots for HIV testing, intravenous blood for HSV-2 testing, and urine for pregnancy testing. Results will be provided within 15 minutes in the context of post-test counseling for HIV and pregnancy. Participants will return within 10 days for HSV-2, and their enrollment visit. Anyone who tests positive for any of these outcomes is ineligible, and will be referred to youth friendly services for clinical care in her area. If the participant’s results are indeterminate, intravenous blood will be used for confirmatory testing using HIV PCR, the results of which will be available within 10 days. She will not be enrolled until the results have been finalized. Participants will also complete a baseline interview (ACASI) at enrollment.

Participants will be randomized at enrollment to one of the two study arms. Details of activities within the two groups are described below, however the main difference is that the participants in the intervention group will also receive vocational training, end of training completion bonus, and career counseling. All other activities will be exactly the same for both groups.

**Prevention for Positives Cohort:** The study procedures for the main study also apply to the new cohort of 50 with the exception that those who positive for pregnancy will still be ineligible while those positive for HIV or HSV-2 will not be excluded. Pregnancy positives will be counseled and referred to the national system for care and follow up.

Specific minor changes that will be made to address the special needs of positives include:

- Revision of counseling scripts and supplemental training for nurses and other staff on issues related to HIV and youth, best clinical practices, confidentiality, stigma and social support, etc.
- Offer volunteer support groups for infected/affected participants, or ensure referral to existing groups in area
- Utilize UZ-UCSF Programme referral protocols for positive study participants to facilitate access to national treatment services.
- Modify data collection tools to assess additional clinicial, behavioral, social and biological outcomes as appropriate.
- Incorporate ‘living positively’ tenants to all participants at Shaz reunions. Examples might include urban gardens and nutrition tips, etc.
- Adding qualitative interviews (and observations of Lifeskills sessions) among both control and interview participants (5 interviews each group, randomly selected, repeated over time) within the new cohort to assess issues such as group dynamics during, especially disclosure, stigma/discrimination and confidentiality.

Outcome measures to be added include:

- Clinical assessment of HIV related opportunistic infections including weight gain/loss
- CD4 counts to assess disease progression and need for referrals for treatment (CDV<250)
- Measures of disclosure, social support, experience of stigma or marginalization at home and in the community through supplemental questionnaires administrated during counseling sessions, qualitative interviews and observations of life skills sessions.

As the intervention and control trainings are designed for an initial group of 25 participants, a training session will begin each time that number of participants is achieved in a given arm. The intervention, which will be delivered in the first year of the study, will consist of five main components: 1) Life Skills education (UZ-UCSF) and basic home-based health care training (Red Cross) (intervention and control arm) 2) Guardian support 3) Choice of vocational training options as determined appropriate and available by SHAZ! guidance counselors (plus stipend/bonus, intervention only); 4) Guidance counseling for social support (intervention arm only) 5) Ongoing SHAZ reunions. There will be one year of follow-up during which time the reunions and study visits will be on-going every six months. (See intervention overview for details).

B. Overview of Intervention

i. Intervention Group

During the first four months of the intervention, participants will participate in an integrated life skills and basic home health care training package, conducted by the UZ-UCSF research team and the Red Cross of Zimbabwe. Training sessions will be delivered to groups of 25 girls living in the same geographical area. The goal of these sessions will be to increase participants’ awareness of risks and opportunities. During this time, the study will provide training and transport fees, and morning tea and breakfast on a weekly basis, based on attendance. To foster social support for the participants, they will be required to meet with a study assigned guidance counselor to discuss vocational options and career growth, and they will be asked to bring in a signed letter of support from their guardians. There will be an orientation for guardians as well. At the end of the life skills and health training sessions, participants will attend a graduation party.

The goal of the next phase of the project is to assess the participants’ readiness to choose and acquire vocational skills. To advance to vocational training, participants will have had to complete at least 80% of all training sessions and all required activities, they will have had to pass the Red Cross course practical exam (allowing for two attempts), and have made at least one visit to the guidance counselor. If they meet these requirements, the guidance counselor will help the participant select an appropriate vocational training option.

Once making her selection, the participant will be placed in one of the three training courses that will take place over a 6-month period with the objective to acquire vocational skills and practice life skills. Based on monthly assessments of attendance, training fees will be paid directly to institutions and participants will receive a stipend for transport and basic food requirements. Fees and stipends will be withdrawn if the participant does not attend at least 80% of the sessions. If participant falls ill or is otherwise unable to attend for legitimate reasons, participants will be allowed to make up sessions. The participant is also required to meet with her guidance counselor at least once. At the end of the training session, if the participant attended at least 80% of the sessions, passed the final exam and made guidance counselor contact she will be eligible for a bonus (equivalent to US$100), contingent upon working with her guidance counselor to develop a plan to use this bonus towards ongoing education, accessing credit or starting up a business using her acquired skills or to secure employment. The participant will have two chances to pass her final examination. At this stage, the guidance counselor will assist the participant in linking to jobs, accessing further training and micro-credit as appropriate. Throughout the course of the study, staff will have ongoing contact with the participant during SHAZ! reunions with her initial life skills class and study visits at alternating 6 month intervals.

ii. Control Group

The control group will have a comparable experience for all aspects of the intervention, except for the vocational training and accompanying monthly fees, stipend and completion bonus, and the personalized guidance counseling. After the first year of the study, the intervention will no longer be delivered. We will follow-up participants from both arms for one additional year, conducting follow-up ACASI interviews and the same biological tests every six months.

To ensure high follow-up rates, at the completion of the participants life skills and health training sessions, intervention and control participants will participate in a graduation ceremony, and then will meet together again in SHAZ reunions every six months, identical to the intervention participants. They will not have access to guidance counseling or vocational training, but will be followed-up for the same amount of time at the same intervals as the intervention participants. Every participant will be provided with condoms and hormonal contraception if requested, and screening and treatment for symptomatic STIs. Referrals for health care will be provided on an as needed basis. Life-skills facilitators/mentors will continue to meet with their peer support groups on a monthly basis for the remainder of the study.

At the end of the study, all control participants who completed a final study visit will be offered the same vocational training that was offered to the intervention participants. The study will cover the fees of the vocational training program for 6 months, but will not provide the transport reimbursement or bonus, or the supportive guidance counseling.

**2. Time:** It will take approximately 15 minutes to explain the study and approximately 30 minutes to get informed consent. The screening interview will take 15 minutes, while the baseline and follow-up interviews will be limited to 30-45 minutes. HIV pre-test counseling will take about 30 minutes, and the time for post-test counseling will depend on the participant’s needs. Blood and urine specimen collection will take ten minutes. Pregnancy counseling will take five to ten minutes.

**3. Study Sites:** Study visits will take place in a family health clinic in Chitungwiza, an urban site-outside of Harare, Zimbabwe.

**G. R**isks/Discomforts

The primary potential risk to participants is a loss of confidentiality. We will minimize this risk by performing training of field staff to ensure full understanding of ethical issues in this type of research and procedures to minimize risk. Consent and data forms will be kept in locked files at the study site. Screening eligibility interviews will be administered using a self-administered paper questionnaire; enrollment and follow-up interview will be conducted using the ACASI system. These computer records will be password-protected to prohibit illicit access. Follow-up and tracking data will also be kept in locked file cabinets physically separate from laboratory data. All personal identifiers will be removed from paper study forms, which will be coded only by numerical identifiers. HSV-2 and HIV (in the case of confirmatory tests sent to the core lab) test requests and results will be transported to and from the study site in sealed envelopes, and specimens will be labeled by study number only. Only the nurse/counselors and lab technicians and study data analysts will have access to test results.

**Prevention for Positives cohort**: As the Shaz study currently has positives enrolled, those who have seroconverted during the study, we feel that issues related to enrolling known seropositives in the study, while sensitive and potentially difficult, are already being managed sensitively and appropriately by our experienced study staff and that an additional group of positives can be integrated equally well, particularly with the training and related modifications listed in Procedures (Section F,1) above.

We will keep confidential all information shared with us, and all results of medical tests to the extent permitted by law, with the exception of knowledge of that which one has moral obligation to report to authorities (such as child abuse). We will minimize losses of confidentiality during follow-up by using only those methods of contact that are acceptable and applicable to participants. Reminder letters will not provide any specific information about the project. Outreach workers will avoid discussing any study specifics when trying to locate participants at home, and will speak only to people – including family members -- to whom participants have granted us permission to speak. For field and phone contact, a code name for the study, known only to the participant, will be used when necessary. When a message is left for the participant at any location, only the code name will be used. If the participant does not have access to a telephone or mailing address, if the letter and phone reminders fail, or if we lack permission to contact the participant by mail, we will try to contact her through a home visit, when permission for such visits has been obtained.

Some participants will feel pain from the needle prick when blood is drawn, and experience a bruise or swelling. We will minimize this risk by hiring nurse/counselors trained in conducting blood draws, and by providing ice to reduce swelling when it occurs. Knowing their HIV, HSV-2 or pregnancy results may cause some participants to worry, or to be stigmatized by those who learn their status. We will minimize this risk by ensuring the confidentiality of results, by providing counseling and support to participants and, if they desire it, their family members or friends, and by providing referrals to support agencies.

H. Treatment and Compensation for Injury

In the event that a participant tests positive for HSV-2, care (for active lesions) will be provided free of charge according to standard of care in Zimbabwe. In the event that a participant tests positive for HIV, the participant and her family (if she desires) will receive ongoing support and counseling through monthly visits by the mobile clinic nurse/counselor, and the participant will be referred to agencies in the area for support services, including medical care. Pregnant participants will be counseled by mobile clinic staff and referred to clinics. In addition, referrals will be made for physical and mental health or other social services, as needed.

**I. Alternatives**

Adolescents can elect not to participate in the study.

**J. Costs to Subject**

There are no costs to participants.

**K. Reimbursement of Subjects**

Participants will be reimbursed for each study visit, and be given a small item worth approximately US$5 to US$10 imprinted with the study logo (such as a T-shirt) upon joining the study. This reimbursement will be in Zimbabwean currency and will be given at the conclusion of each data collection visit, and it will be equivalent to approximately US$5. No reimbursement will be provided for trainings or weekly meetings, however food and transport will be provided when appropriate.

Participants in the intervention group who successfully complete their vocational training and pass their final exam will receive a bonus equivalent to $100 US. This bonus is to serve both as an incentive to attend and do well in their training, and to help them do well in their chosen field after completion of the training. The bonus is not to be used for personal spending. Participants will be required to work with a guidance counsellor to come up with a detailed plan for how they will use this money for ongoing education, accessing credit, or for purchasing capital equipment for a business or income generating project in order to be eligible to receive this bonus.

**L. Confidentiality of Records**

All study participants will receive a unique study identification number (ID) and barcode that will be recorded on all paper forms, such as the screening/eligibility questionnaire and laboratory forms. This study ID will also be used in the ACASI system. One electronic file that links participants’ names and study ID numbers will be maintained by the field director and will be stored in encrypted form on a password protected, secure computer. The encrypted list and private encryption key will be stored separately off-site, and the key communicated to on-site study staff, if needed, via telephone. Paper consent forms, contact information sheets, and other paper data collection instruments will be stored in a locked file cabinet located in the project offices. Paper study forms for eligibility and lab results will be transmitted via email using Internet-capable fax machines to the primary data entry and management system. Forms will be converted directly to ASCII-formatted data files using the DataFax system (www.datafax.com). Datasets from ACASI-based interviews will be sent to the data coordinating center via email in compressed form. A study-specific web site will be created including publicly accessible study information and a restricted access section including project documentation and reports. A full backup is performed daily (except Saturday and Sunday). Copies of backup tapes are regularly archived at a remote location. Original data disks will also be stored in a locked file cabinet at the study site. Once data have been saved in the primary database, interviews will be removed from the interviewing field computers, and diskettes will function as a secondary data back-up system. On a weekly basis, the data from the disks will be accumulated into a primary data file on a password-protected computer.

**5. Qualification of Investigators**

The Principal Investigator, Nancy Padian, has conducted research on STI/HIV risk in high-risk populations (including adolescents) for close to twenty years. For the last four years, Megan Dunbar has directed the adolescent livelihoods projects in Zimbabwe. Dr. Sue Laver, the Zimbabwean Principal Investigator, offers expertise in behavioral science as it relates to HIV prevention. Stephen Shiboski, the project senior statistician, has extensive experience analyzing STI/HIV risks and the likelihood of transmission. Catherine Maternowska has conducted quantitative and qualitative research examining reproductive health and rights for over 15 years, including research on gender and power issues as they affect STIs, and has worked extensively with the Pulerwitz scale, upon which we will base much of our data collection.

**6. Bibliography**

1. SHAZ, *Shaping the Health of Adolescents in Zimbabwe Project, 2000-2002*. 2001: Harare, Zimbabwe.

2. Parker, R., Easton, D., Klein CH., *Stuctural barriers and facilitators in HIV prevention: a review of international research.* AIDS, 2000. **14**: p. S22-32.

3. de Bruyn, M., *Women and AIDS in developing countries.* Soc Sci Med, 1992, February. **34**(3): p. 249-62.

4. Sachs, A., *AIDS orphans: Africa's lost generation.* World Watch, Sept-Oct 1993. **6**(5): p. 10.

5. UNAIDS, *Epidemiological fact sheet on HIV/AIDS and sexuallly transmitted diseases*. 2000, UNAIDS/WHO Working Group: Geneva.

6. MOHCW, *National Survey Of HIV and Syphillis Prevalence Among Women Attending Antenatal Clinics in Zimbabwe, 2000*. 2000, Ministry of Health and Child Welfare - Zimbabwe: Harare.

7. Mbizvo, M., *Effects of intervention strategies on selected aspects of teenage reproductive health.* 1998.

8. JHU, *Population Reports*. 2001, Johns Hopkins University.

9. Cowan, F., *Personal Communication*,, M. Dunbar, Editor. 2001: Harare, Zimabwe.

10. Obasi, A., et al., *Antibody to herpes simplex virus type 2 as a marker of sexual risk behavior in rural Tanzania.* J Infect Dis, 1999. **179**(1): p. 16-24.

11. Latif, A.S., et al., *Risk factors for gonococcal and chlamydial cervical infection in pregnant and non-pregnant women in Zimbabwe.* Central African Journal of Medicine, 1999. **45**(10): p. 252-8.

12. DHS, *Knowledge of STIs and AIDS, Risk Awareness, and Condom Use*. Zimbabwe Further Analysis, ed. M. Mbivzo, et al. 1997, Maryland: Macro International.

13. UNAIDS, U., WHO, *Update: Zimbabwe, Epidemiological Fact Sheets on HIV/AIDS & Sexually Transmitted Infections*. 2002.

14. Buve, A., et al., *Ethics of mass STD treatment.* Lancet, 2000. **356**(9235): p. 1115-1116.

15. Weiss, H., Buve A. et al, *The epidemiology of HSV-2 infection and its association with HIV infection in four urban African populations.* AIDS, 2001. **15**(Suppl: 4): p. S97-108.

16. Holmberg, S.D., et al., *Prior herpes simplex virus type 2 infection as a risk factor for HIV infection.* Journal of the American Medical Association, 1988. **259**(7): p. 1040-50.

17. Latif, A., *A report on a study to determine the aetiology and pattern of STD amongst men and women presenting to health centres in Harare, Zimbabwe, and to determine risk factors for cervicitis among symptomatic and asymptomatic women*. 1995, University of Zimbabwe: Harare.

18. Wasserheit, J.N., *Epidemiological synergy. Interrelationships between human immunodeficiency virus infection and other sexually transmitted diseases.* Sexually Transmitted Diseases, 1992. **19**(2): p. 61-77.

19. Wasserheit, J.N. and S.O. Aral, *The dynamic topology of sexually transmitted disease epidemics: implications for prevention strategies.* J Infect Dis, 1996. **174 Suppl 2**: p. S201-13.

20. Foster, G. and J. Williamson, *A review of current literature on the impact of HIV/AIDS on children in sub-Saharan Africa.* Aids, 2000. **14**(Suppl 3): p. S275-84.

21. Foster, G., *Supporting community efforts to assist orphans in Africa.* N Engl J Med, 2002. **346**(24): p. 1907-10.

22. UNAIDS/WHO, *AIDS epidemic update.* 2001: p. 1-22.

23. Sengando, J. and J. Nambi, *The psychological effect of orphanhood: a study of orphans in Rakai District.* Health Transition Review, 1997. **10 (suppl)**: p. 105-24.

24. Kadenge, L., *Macroeconomic Indicators for Zimbabwe - an update*. 2002, Zimbabwe Economics Society: Harare. p. 1.

25. Elson, D. and R. McGee, *Gender equality, bilateral program assistance and structural adjustment: Policy and procedures.* World Development, 1995. **23**(11): p. 1987-1994.

26. Parker, R.G., *Empowerment, community mobilization and social change in the face of HIV/AIDS.* Aids, 1996. **10 Suppl 3**(2): p. S27-31.

27. Mathur, S., A. Malhotra, and M. Mehta, *Adolescent girls' life aspirations and reproductive health in Nepal.* Reproductive Health Matters, 2001. **9**(17): p. 91-100.

28. Nzyuko, S., et al., *Adolescent sexual behavior along the Trans-Africa Highway in Kenya.* Aids, 1997. **11**(SUPP1): p. S21-S26.

29. Calves, A.-E., Gretchen T. Cornwell, and Parfait Eloundou Enyegue, *Adolescent sexual activity in sub-Saharan Africa: Do men have the same strategies and motivations as women?* Working Paper AD96-04. Population Research Institute, Pennsylvania State University, 1996.

30. Laga, M., et al., *To stem HIV in Africa, prevent transmission to young women.* AIDS, 2001. **15**: p. 931-934.

31. PRB, *Women of our World.* Population Reference Bureau, 1998. **Washington, DC**: p. PRB.

32. Irvin, A., *Taking steps of courage: Teaching adolescents about sexuality and gender in Nigeria and Cameroon.* New York: International Women's Health Coalition, 2000.

33. Weiss, E., Daniel Whelan, and Geeta Rao Gupta, *Vulnerability and opportunity: Adolescents and HIV/AIDS in the developing world.* Washington, DC: International Center for Research on Women, 1996.

34. Wood, K., Fidelia Maforah, and Rachel Jewkes, *"He forced me to love him": putting violence on adolescent sexual health agendas.* Social science and medicine, 1998. **47**(2): p. 233-242.

35. Fuglesang, M., *Lessons for life - Past and present modes of sexuality education in Tanzanian society.* Social Science & Medicine, 1997. **44**(8): p. 1245-1254.

36. Webb, D., *HIV and AIDS in Africa*. 1997, London: Pluto Press. xiii, 258.

37. Gage, A.J. and D. Meekers, *Sexual Activity before Marriage in Sub-Saharan Africa.* Social Biology, 1994. **41**(1-2): p. 44-60.

38. Haram, L., *Negotiating sexuality in times of economic want: The young and modern Meru women*, in *Young people at risk: Fighting AIDS in Northern Tanzania*, P.M.B. Knut-Inge Klepp, and Aud Talle, eds., Editor. 1995, Scandinavian University Press: Oslo.

39. Komba-Malekela, B.a.R.L., *Looking for men*, in *Chelewa, chelewa. The dilemma of teenage girls*, e. Zubeida Tumbo-Masabo and Rita Liljestrom, Editor. 1994, The Scandinavian Institute of African Studies.

40. PRB, *Sexual violence against young women. World Population Fact Sheet*, in *The World's Youth 2000*. 2001, Population Reference Bureau: Washington, Dc.

41. Pulerwtiz, J., Gortmaker, S.L. et al, *Measuring Sexual Relationship Power in HIV/STD Research.* Sex Roles, 2000. **42**(7/8): p. 637-660.

42. Luke, N., *Cross-generational and Transactional Sexual Relations in Sub-Saharan Africa:*

*A Review of the Evidence on Prevalence and Implications for Negotiation of Safe Sexual Practices for*

*Adolescent Girls.* 2001: p. 1-54.

43. Wojcicki, J.M. and J. Malala, *Condom use, power and HIV/AIDS risk: sex-workers bargain for survival in Hillbrow/Joubert Park/Berea, Johannesburg.* Social Science & Medicine, 2001. **53**(1): p. 99-121.

44. Barnes, C., *The relationship between microfinance and households coping with illness and death In Zimbabwe:*

*an exploratory study.* 2001: p. 1-26.

45. Rodrigues-Garcia, R., James Macinko and William Waters, *Microenterprise Development for Better Health Outcomes*. 2001, Westport, CT: Greenwood Press.

46. Pulerwitz, J., S.L. Gortmaker, and W. DeJong, *Measuring sexual relationship power in HIV/STD research.* Sex Roles, 2000. **42**(7-8): p. 637-660.

47. Luke, N., *A review of evidence on prevalence and implications for negotiation of safe sexual practices for adolscent girls*. 2001, International centre for research on women: Philadelphia.

48. Hashemi, S.M., S.R. Schuler, and A.P. Riley, *Rural Credit Programs and Womens Empowerment in Bangladesh.* World Development, 1996. **V24**(N4): p. 635-653.

49. Schuler, S.R., S.M. Hashemi, and A.P. Riley, *The influence of women's changing roles and status in Bangladesh's fertility transition: Evidence from a study of credit programs and contraceptive use.* World Development, 1997. **25**(4): p. 563-575.

50. Amin, R., et al., *Impact of poor women's participation in credit-based self-employment on their empowerment, fertility, contraceptive use, and fertility desire in rural Bangladesh.*, in *Presented at the Annual Meeting of the Population Association of America, Miami, Florida, May 5-7, 1994*. 1994.

51. Khandker, S.a.B.K., *The Bangladesh Rural Advancement Committee's Credit Programs: Performance and Sustainability. Discussion Papers #324*. 1996, World Bank.: Washington, DC.

52. Schuler, S.R., et al., *Credit programs, patriarchy and men's violence against women in rural Bangladesh.* Soc Sci Med, 1996. **43**(12): p. 1729-42.

53. Fawole, I.O., et al., *A school-based AIDS education programme for secondary school students in Nigeria: a review of effectiveness.* Health Educ Res, 1999. **14**(5): p. 675-83.

54. Welbourn, A., *Gender, Sex and HIV: how to address issues that no-one wants to hear about*, in *Tant qu'on a la sante*. 1999, Geneva Symposium - DDC, UNESCO and IUED: Geneva. p. 195-227.

55. UNICEF, *Growing up in Zimbabwe*. 1999: Harare.

56. Group, I.G.W., *Guide for Incorporating Gender Considerations in USAID’s Family Planning and Reproductive Health - RFAs RFPs*. 2000, USAID: Washington, DC.

57. Hubley, J. and L. Fransen, *Health education and sexually transmitted diseases.* Trop Doct, 1996. **26**(3): p. 121-5.

58. Caceres, C.F., et al., *Evaluating a school-based intervention for STD/AIDS prevention in Peru.* J Adolesc Health, 1994. **15**(7): p. 582-91.

59. Snow, D.R. and T.F. Buss, *Development and the role of microcredit.* Policy Studies Journal, 2001. **29**(2): p. 296-307.

60. Chinake, H., Dunbar M. et al, *Intergenerational sex among adolescents in Zimbabwe*. 2002, AIDS 2002 Conference: Barcelona, Spain.

61. Dunbar, M., Chinake, H. et al, *Economic and social factors related to HIV risk among adolescent females in Zimbabwe*. 2002, AIDS 2002 Conference: Barcelona.

62. Emerson, R., *Exchange Theory, Part I: A Psychological Basis for Social Exchange; Exchange Theory, Part II: Exchange Relations and Network Structures*, in *Sociological Theories in Progress*, M.Z.J. J. Berger, B. Anderson, Editor. 1972, Houghton Mifflin Company: Boston, Atlanta, Geneva (Ill.), Dallas, Palo Alto. p. 38-87.

63. Connell, R., *Theorizing gender.* Sociology, 1985. **19**: p. 260-272.

64. Emerson, R., *Power, equity, and commitment in exchange networks.* American Sociological Review, 1978. **43**(721).

65. Wingood, G.M., *Partner Influences and gender-related factors associated with noncondom use among young adult African American women.* American Journal of Community Psychology, 1998. **26**(1): p. 29-51.

66. Wingood, G. and R. DiClemente, *Application of the theory of gender and power to examine HIV-related exposures, risk factors, and effective interventions for women.* Health Education and Behavior, 2000. **27**: p. 539-65.

67. Clark, H., *Some Issues for Consideration for the Workshop on Microfinance and HIV/AIDS*. 1999, UNAIDS Workshop.

68. DAI, *The MBP Reader on Microfinance and HIV/AIDS: First Steps in Speaking Out*. 2000, Africa Regional Microcredit Summit: Harare, Zimbabwe.

69. World Health Organization, S.a.E.R.G., *Preparing a Project Proposal, Guidelines and Forms (Third Edition)*. 2000.

70. Bennell, P. 2000. *Improving Youth Livelihoods in sub-Saharan Africa*: A review of policies and programs with particular emphasis on the link between sexual behavior and economic well-being. Report to the International Development Center (IDRC).

71. UNAIDS, *Table of country-specific HIV/AIDS estimates and data*. 2000, www.unaids.org.

72. Collett-White, C., L. David, *Dream, Dare, Do: Girl Guides and Girl Scouts learn ways to improve nutrition*. 1999, World Association of Girl Guides and Girl Scouts (WAGGGS) 30th World Conference: Dublin, Ireland.

73. Welbourn, A., *Stepping Stones: A Training Package on HIV/AIDS, Communication and Relationship Skills*. 1999.

74. Fuglesang, A. and D. Chandler, *Participation as Process—Participation as Growth*. 1995, Grameen Trust: Dhaka, Bangladesh.

75. Cowan, F.M., *Testing for type-specific antibody to herpes simplex virus - implications for clinical practice.* Journal of Antimicrobial Chemotherapy, 2000. **V45**(SUPPT3): p. 9-13.

76. Gregson, S. and G.P. Garnett, *Contrasting gender differentials in HIV-1 prevalence and associated mortality increase in eastern and southern Africa: artefact of data or natural course of epidemics'.* Aids, 2000. **14**(SUPP3): p. S85-S99.

77. Hayes, R.J. and S. Bennett, *Simple sample size calculation for cluster-randomized trials.* Int J Epidemiol, 1999. **28**(2): p. 319-26.

78. Wawer, M.J., et al., *Control of sexually transmitted diseases for AIDS prevention in Uganda: a randomised community trial.* Lancet, 1999. **353**(9152): p. 525-535.

79. Rompalo A, H.N., Colletta L, et al., *A new accurate point of care test kit, POCkit, for the Diagnosis of HSV-2 Infection.* Diagnology, 1999.

80. Heise, L.L. and C. Elias, *Transforming aids prevention to meet women's needs: a focus on developing countries.* Soc Sci Med, 1995. **40**(7): p. 931-43.

81. Edgcomb, E.a.L.B., *Social Intermediation and Microfinance Programs: A Literature Review.*, in *Microenterprise Best Practices*. 1998, Development Alternatives, Inc.: Washington, DC.

82. Cheston, S.a.L.R., *Measuring transformation: assessing and improving the impact of microcredit*. 1999, Microcredit Summit: Abidgan, Cote d'Ivoire.

83. Rankin, K., *Governing development: neoliberalism, microcredit, and rational economic woman.* Economy and Society, 2001. **30**(1): p. 18-37.

84. Wingood, G. and R. DiClemente, *Cultural, gender and psychosocial influences on HIV-related behavior of African American female adolescents: Implications for the development of tailored prevention programs.* Ethnicity and Disease, 1992(3): p. 381-388.

85. Diggle, P., K. Liang, and S. Zeger, *Analysis of Longitudinal Data*. 1994, Oxford: Oxford University Press.

86. Breiman, L., et al., *Classification and Regression Trees*. 1984, Monterey, CA: Wadsworth and Brooks.

87. Grierson, J. *Youth, Enterprise, Livelihoods and Reproductive Health: How Can Small Enterprise and Sustainable Livelihoods Help Address the HIV/AIDS Crisis in Africa*, 2000.

88. Rosen, J. *Youth Livelihoods and HIV/AIDS*, YouthNet InFocus, Family Health International, 2001

89. Esim, S., and A. Malhotra et al.. *Making* *It Work: Linking Youth Reproductive Health and Livelihoods.* International Center for Research on Women, Washington, D.C. 2001. http://www.icrw.org/docs/Makingitwork.pdf

90. Bloome, Anthony. *Youth Livelihoods and HIV/AIDS: Literature Review and Policy Recommendations.* Report to International Labor Organization, 2005, (unpublished).

Appendix 1: Study Flow Chart

Appendix 2: Intervention Framework
